# Extensor Digitorum Brevis Manus: A Comprehensive Review of this Variant Muscle of the Dorsal Hand

**DOI:** 10.7759/cureus.1568

**Published:** 2017-08-15

**Authors:** Rafik Shereen, Marios Loukas, R. Shane Tubbs

**Affiliations:** 1 Department of Anatomical Sciences, St. George's University School of Medicine, Grenada, West Indies; 2 Department of Anatomical Sciences, St. George's University School of Medicine, Grenada, West Indies; 3 Neurosurgery, Seattle Science Foundation

**Keywords:** extensor digitorum brevis manus, management, variants, excision

## Abstract

The extensor digitorum brevis manus (EDBM) is a variant muscle located on the dorsum of the hand. This variant of the fourth compartment has often eluded preoperative diagnosis and led to unnecessary repeat visits to the operating room owing to its lack of notoriety. As a result, we aim to review the literature concerning the EDBM with respect to its embryology, comparative anatomy and variants, and clinical significance in an attempt to increase awareness and help in preoperative diagnosis and management. A total of 21 articles were reviewed. The results show that the EDBM often goes underdiagnosed and is frequently discovered incidentally in the operating room or in cadaveric dissections. There are multiple variations of the EDBM that, in certain instances, dictate the correct method of management. While there has been a reversal of opinions on which technique is the most popular at relieving symptoms caused by the EDBM, current arguments stand for retinacular release only when the EDBM serves as a sole extensor for one of the indices. In other instances where this is not the case, surgical excision of the EDBM proved to be the most effective at relieving symptoms.

## Introduction and background

**Figure 1:**
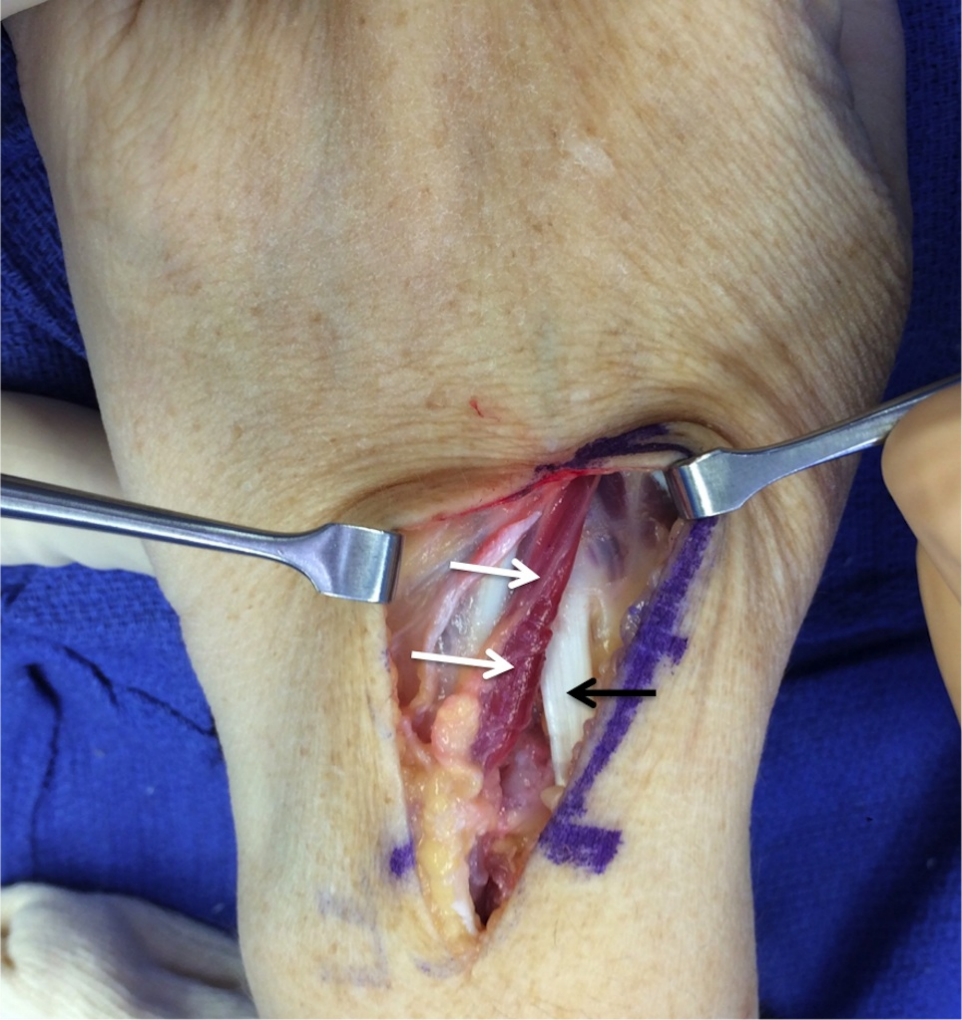
Example of extensor digitorum brevis manus muscle (white arrows) found during surgery of the left hand. For reference, note the tendon of the extensor carpi radialis longus muscle (black arrow).

A variant of the extensors located in the dorsum of the hand is the extensor digitorum brevis manus (EDBM) (Figure 1). This muscle was first described by a German-born Dutch anatomist, Bernhard Albinus, who used the term extensor brevis digiti indicis vel medii [1]. Since then the EDBM has been given multiple synonyms, but ‘extensor digitorum brevis manus,’ first used by Macalister in 1875 [2], has become the most commonly accepted term.

A recent systematic review of twenty-five studies reporting the true cadaveric prevalence of the EDBM revealed a pooled prevalence estimate (PPE) of 2.3% [3]. Although not an uncommon variant, the EDBM has proved a difficult preoperative diagnosis for patients presenting with chronic dorsal wrist pain and is usually discovered during surgery [4-5]. Yammine considered the diagnosis of the EDBM to be: “One of those conditions where one should think of it to make the diagnosis; it should be included in the differential diagnosis of ‘dorsal ganglions’” [3]. This variant has therefore been researched in attempts to increase awareness and help bring to light the preoperative differential when diagnoses are being considered.

## Review

Embryology

The precursor muscle that gives rise to the extensors of the forearm differentiates into superficial, deep, and radial portions. While the superficial portion has remained relatively stable throughout evolution, comparative anatomy studies have shown that the deep portion has undergone significant changes, giving rise to the variants seen amongst primates [5]. Therefore, some researchers have inferred that the EDBM arises from the deep, unstable portion of the forearm extensor precursor muscle [3]. Others have suggested that it represents the failed proximal migration of the undifferentiated extensor muscle mass of the hand, similar to an atavistic structure [6-7].

Comparative anatomy and variants

Although there are variants, multiple studies have reported the most common origin for the EDBM to be inside the radiocarpal joint capsule, attaching either to the dorsal metacarpal surface, the distal end of the radius, or the proximal portion of the radiocarpal ligament [3, 8-9]. The distal insertion of the EDBM is often joined to the extensor indicis proprius (EIP), which serves to extend the index finger, and the two muscles share the same innervation and blood supply from the posterior interosseous nerve and anterior interosseous artery, respectively [3]. Ogura, et al. revealed that ten out of twenty human upper limbs lacking the EIP had an EDBM, suggesting that the EDBM could serve to compensate for the lack of an EIP and, in such instances, serve as the only extensor for the index finger, justifying the use of the term extensor indicis brevis [9].

Ogura, et al. classified the different variants according to their insertion in relation to the EIP. They were classified into three groups: group I had an EIP missing with the EDBM attached to the index finger on the dorsal aponeurosis; group II had an EDBM attached to the index finger along with the EIP; and group III had it attached to the long finger, aiding (though not solely) in extension of the middle finger [9]. Group II was further classified into three subgroups depending on how the EDBM and EIP interacted: subgroup A was defined as an EDBM connected to a shortened or vestigial EIP confluent with the belly of the EDBM inserted into the index finger; subgroup B was defined as an EDBM inserted along the ulnar side of the EIP; and in subgroup C, the EDBM was inserted into the distal end of the EIP [9]. Another attempt to classify the variants on the basis of their distal insertion was made by Yoshida [10], but this classification scheme is not as commonly used, so it will not be discussed in detail in this review.

Previous studies have led to different opinions concerning the bilateral occurrence of EDBM and familial inheritance. In their Japanese study of roughly 600 cadavers, Ogura, et al. cited a bilateral occurrence rate of about 50% for patients suffering from EDBM [9]. In a 2014 meta-analysis, which included Ogura’s data, the incidence of bilateral EDBM was 26.3% [3]. Whether there is a genetic/familial component to the occurrence of EDBM is also still debatable. As previously mentioned, there are multiple accounts of the origin of the EDBM, one of which emanates from data on EDBM being diagnosed within relatives, suggesting some type of familial inheritance. For example, in their study of 3404 adults, Gama, et al. identified 38 individuals with the EDBM, three of whom had relatives with the same condition [11]. More research should establish whether this number is significant and suggests some type of familial inheritance. It is also important to note that many clinical case reports have revealed more manual laborers with dominant hand EDBM pathology, suggesting a non-familial pattern of occurrence. Interestingly, a case of two brothers both suffering from non-dominant hand EDBM has been reported [12].

Clinical significance

While the EDBM can be found incidentally during surgery or in a cadaveric dissection, few cases have involved patients reporting symptoms prior to the discovery of this anomaly of a dorsal hand muscle. In a study of more than 300 clinical and cadaveric case reports, few patients reported symptoms prior to presentation [13].

When symptoms are present, they typically consist of chronic pain on the dorsum of the wrist. In 1999, Hayashi, et al. coined the term “fourth compartment syndrome,” which attributed the etiology of chronic dorsal wrist pain to causes within the extensor retinaculum [14]. They listed five possible causes: EDBM, ganglion involvement, abnormal extensor indicis muscle, tenosynovitis, and anomaly or deformity of the carpal bones [14]. The EDBM has been thought to contribute pain in fourth compartment syndrome as a result of inflammation and hypertrophy [3], but to complicate things further, cases of the EDBM having a ganglion associated or embedded in the muscle itself have been reported [9, 15]. In Ogura’s study, 17 ganglia were associated with 68 cases of EDBM. As previously mentioned, the EDBM increases edema on the dorsum of the hand, often leading to a preoperative misdiagnosis of a synovial cyst [16]. While the exact etiology of chronic pain due to EDBM is still in question, this should not deter the physician from keeping this differential in mind at the presentation of symptoms.

According to Ross, et al., aggravating factors that can potentially contribute to the expression of EDBM symptoms include manual labor and hand dominance [17]. Although magnetic resonance imaging (MRI) scans can aid in the diagnosis, they do not always help if the radiologist fails to consider the EDBM as a differential [18]. Also, while electrophysiological studies can help distinguish cysts or tumors from EDBM, the physician again needs to consider EDBM as a differential in order to perform those studies. As per Yammine’s declaration that EDBM should be included in the differential diagnosis of dorsal ganglia, if the physician is not thinking of it, it is often missed [3, 18].

With most cases being incidental findings during surgery or cadaveric dissections, treatment is not warranted until chronic pathology develops. When symptomatic, the treatment for EDBM usually involves surgical excision of the retinaculum or removal of the muscle (partial/debridement or complete). While some literature recommends conservative treatment, data on the effectiveness of conservative treatment are limited. Most of the literature explored agreed on surgical intervention for symptomatic EDBM. It is worth noting that one case report in which EDBM pathology was treated with botulinum toxin showed significant improvement [19], but more powerful studies are required before this finding can be validated statistically.

Since its discovery, algorithms for different surgical procedures for the EDBM have been developed, but opinions vary. Initially, complete excision of the muscle seemed to be the preferred method of treatment. Then during the 1980s, when EDBM variants were more clearly classified, retinacular release gained popularity. Patel, et al. proposed that the primary treatment should be retinacular release, especially when the EDBM serves to compensate for the extensor indicis proprius, if the muscle is electrophysiologically and histopathologically normal, or if it can be used successfully as a motor to restore function to the extensor pollicis longus [20]. In cases where retinacular release fails, or upon the patient’s wish for cosmetics, the muscle belly should be excised as the definitive treatment [20].

Ogura, et al. also proposed a logical algorithm based on their classification scheme. For types of EDBM where the EIP was absent or extension of the index finger relied more on the EDBM (groups I and IIA), retinacular division or partial resection was recommended. For variants where the EIP was not absent and there was less compensation for extension of the index finger (groups IIB, IIC, and III), complete excision of the EDBM belly was recommended.

Yammine’s review simply stated that division of the extensor retinaculum yields good results in cases of muscular hypertrophy, but for complete resolution of symptoms, excision of the belly yields the best results. However, given the different variations in insertions and function and the difficulty of ascertaining a preoperative diagnosis, others have indicated that the matter is more complicated.

A case report from 2015 highlights the elusiveness of the EDBM and a logical approach to intraoperative decision-making with regard to decompression versus excision (partial debridement or total) [21]. Waterman highlights how the EDBM can be confused with other pathologies, especially when trauma aggravates the symptoms and how imaging can be misleading prior to surgical intervention. The MRI of this patient suggested some sort of scaphoid pathology, but the EDBM was found incidentally and evaluated in the operating room. In his description of the operation, Waterman traced the tendon insertion and origin sites to determine the mechanical role of the EDBM. Like Ogura and Patel, Waterman emphasized surgical excision over debridement or retinacular release if the EDBM was not a sole extensor for the index [9, 20-21]. The patient underwent total excision and recovered completely. Perhaps this method could be applied to imaging studies. More research should point towards improving imaging studies for anomalous muscle bodies by developing a system of tracking and tracing the insertions and origins. Given this and an increase in clinical awareness, perhaps we could improve the preoperative diagnosis rate and decrease the number of repeat trips to the operating room. Although not a powerful study, Waterman’s approach could pave the way for a larger scale study to determine an effective algorithm for surgical management.

## Conclusions

The EDBM represents a variant of the dorsal extensor muscles of the hand. Since its discovery, it has evaded diagnosis owing to lack of clinical awareness, difficulty of detecting it by imaging, and similarities to other wrist pathologies. The etiology of EDBM has yet to be established. However, there is an increasing amount of literature on this variation, its associations with other wrist pathologies, and its management. While experts agree that surgical management is most effective at relieving symptoms, opinions differ on the correct surgical approach to treating symptomatic EDBM. Over the years there seems to have been a dynamic reversal of preferred technique: decompression versus excision. The main argument for reticular decompression is that it is a simpler approach with fewer complications, and this should be the preferred method when the EDBM is the sole extensor. However, many patients present months after surgery with recurring symptoms and require a second surgery. Complete excision prevents unnecessary second operations and has proved most effective in relieving symptoms. The development of innovative imaging techniques and more research on clinical outcomes of the different surgical approaches could prove useful for determining EDBM preoperatively and for ensuring better patient care.
